# Mechanical Strain Alters Cellular and Nuclear Dynamics at Early Stages of Oligodendrocyte Differentiation

**DOI:** 10.3389/fncel.2018.00059

**Published:** 2018-03-06

**Authors:** Ekta Makhija, Anna Jagielska, Lena Zhu, Alexander C. Bost, William Ong, Sing Y. Chew, G. V. Shivashankar, Krystyn J. Van Vliet

**Affiliations:** ^1^BioSystems and Micromechanics Interdisciplinary Research Group, Singapore-MIT Alliance for Research and Technology, CREATE, Singapore, Singapore; ^2^Department of Materials Science and Engineering, Massachusetts Institute of Technology, Cambridge, MA, United States; ^3^Department of Biological Engineering, Massachusetts Institute of Technology, Cambridge, MA, United States; ^4^Department of Aeronautics and Astronautics, Massachusetts Institute of Technology, Cambridge, MA, United States; ^5^NTU Institute for Health Technologies (Health Tech NTU), Interdisciplinary Graduate School, Nanyang Technological University, Singapore, Singapore; ^6^School of Chemical and Biomedical Engineering, Nanyang Technological University, Singapore, Singapore; ^7^Lee Kong Chian School of Medicine, Nanyang Technological University, Singapore, Singapore; ^8^Mechanobiology Institute, Singapore, Singapore; ^9^The FIRC Institute of Molecular Oncology, Milan, Italy

**Keywords:** oligodendrocyte differentiation, strain, nuclear dynamics, cell migration, microtubules

## Abstract

Mechanical and physical stimuli including material stiffness and topography or applied mechanical strain have been demonstrated to modulate differentiation of glial progenitor and neural stem cells. Recent studies probing such mechanotransduction in oligodendrocytes have focused chiefly on the biomolecular components. However, the cell-level biophysical changes associated with such responses remain largely unknown. Here, we explored mechanotransduction in oligodendrocyte progenitor cells (OPCs) during the first 48 h of differentiation induction by quantifying the biophysical state in terms of nuclear dynamics, cytoskeleton organization, and cell migration. We compared these mechanophenotypic changes in OPCs exposed to both chemical cues (differentiation factors) and mechanical cues (static tensile strain of 10%) with those exposed to only those chemical cues. We observed that mechanical strain significantly hastened the dampening of nuclear fluctuations and decreased OPC migration, consistent with the progression of differentiation. Those biophysical changes were accompanied by increased production of the intracellular microtubule network. These observations provide insights into mechanisms by which mechanical strain of physiological magnitude could promote differentiation of progenitor cells to oligodendrocytes via inducing intracellular biophysical responses over hours to days post induction.

## Introduction

Generation and repair of myelin, the protective lipid membrane that oligodendrocytes wrap around axons in the central nervous system (CNS), is critical for neuron survival and maintenance of neurological functions (Sherman and Brophy, 2005; Nave and Werner, 2014). Myelin disorders can lead to permanent loss of neurons and severe neurological disabilities, and are hallmarks of demyelinating diseases such as multiple sclerosis (Franklin and ffrench-Constant, 2008; Fancy et al., 2011). Our collective understanding of differentiation from oligodendrocyte progenitor cells (OPC) into adult oligodendrocytes that subsequently myelinate axons remains too limited to successfully stimulate myelin repair. Thus, further exploration is required to quantify how extracellular environment regulates these processes *in vitro* and *in vivo*.

Mechanical cues are understood increasingly as essential components of the oligodendrocyte environment that modulate phenotypic maturation and myelin production. We and others have demonstrated that mechanical factors present *in vivo*, such as stiffness of brain tissue and mechanical strains associated with axon growth or swelling, can promote biological processes including oligodendrocyte differentiation (Rosenberg et al., 2008; Kippert et al., 2009; Jagielska et al., 2012, 2017; Arulmoli et al., 2015; Hernandez et al., 2016; Lourenço et al., 2016; Urbanski et al., 2016; Shimizu et al., 2017). We showed previously that physiological ranges of mechanical tensile strain (termed static strain, maintained at constant magnitude of 10%) accelerated the differentiation of oligodendrocytes by inducing global changes in gene expression (Jagielska et al., 2017). Such extracellular strain resulted in changes of histone acetylation within the nucleus, followed temporally by changes in gene expression (recorded at 24 h of applied strain) consistent with OPC differentiation.

In the present work, we focused on visualizing and understanding how such strain is transduced to OPCs to promote differentiation, with a focus on intracellular biophysical responses during the first 48 h of applied strain. We observed through time-lapsed image analysis that nuclear dynamics were reduced significantly within strained cells at 24 h post-induction of differentiation. Such reduced dynamics of the nuclear membrane are consistent with trends observed in other (stem) cell types during differentiation, as chromatin within the nucleus transitions from a dynamic state characteristic of stem cells to a phenotypically committed state (Dahl et al., 2008; Shivashankar, 2011; Mendez and Janmey, 2012; Graham and Burridge, 2016). We further demonstrated that this strain-induced reduction of nuclear dynamics correlated with reduced cell migration and increased microtubule production. Together, these findings demonstrate how the external mechanical cue of static strain promotes intracellular responses that could facilitate oligodendrocyte differentiation.

## Materials and methods

### Ethics statement

All animal procedures were approved by the Nanyang Technological University Institutional Animal Care and Use Committee (IACUC).

### Cell culture and media

Oligodendrocyte progenitor cells (OPCs, 95% pure cell population as verified by NG2 staining Figure S2) were isolated according to previously reported protocols (McCarthy and de Vellis, 1980). Briefly, cortices from P0-P2 SD rats were isolated and dissociated with papain, L-cysteine and DNase I for 60 min at 37°C before plating on Poly-D-lysine coated T75 flask. Mixed glial culture from these cortices were maintained in DMEM High Glucose (Gibco 11995) supplemented with 10% fetal bovine serum (FBS, Gibco) and 1% penicillin/streptomycin (Gibco) for 9–11 days. OPCs were separated from the mixed glial culture by overnight shaking on an orbital shaker. Thereafter, OPCs were further purified by differential adhesion on untreated polystyrene dish for 25 min to remove contaminating microglia and astrocytes. OPCs were then maintained in a progenitor state in DMEM (Invitrogen) with SATO's modification [5 μg/ml insulin, 50 μg/ml holo-transferrin, 5 ng/ml sodium selenate, 16.1 μg/ml putrescine, 62 ng/ml progesterone, 0.1 mg/ml bovine serum albumin (BSA), 0.4 μg/ml Tri-iodothyroxine (T3), 0.4 μg/ml L-Thyroxine (T4)] plus 10 ng/ml PDGF-A and 10 ng/ml FGF2 (Peprotech) (proliferation medium). To induce differentiation, OPCs were cultured in SATO's medium without FGF2 and PDGF-A and with 0.5% fetal bovine serum (FBS, Gibco) (differentiation medium).

Proliferation Medium comprised: DMEM + SATO's components (including T3,T4) + FGF + PDGF. Differentiation Medium comprised: DMEM + SATO's components (including T3, T4) + 0.5% FBS.

### Fabrication and functionalization of elastomeric plates

**Figure 1:**
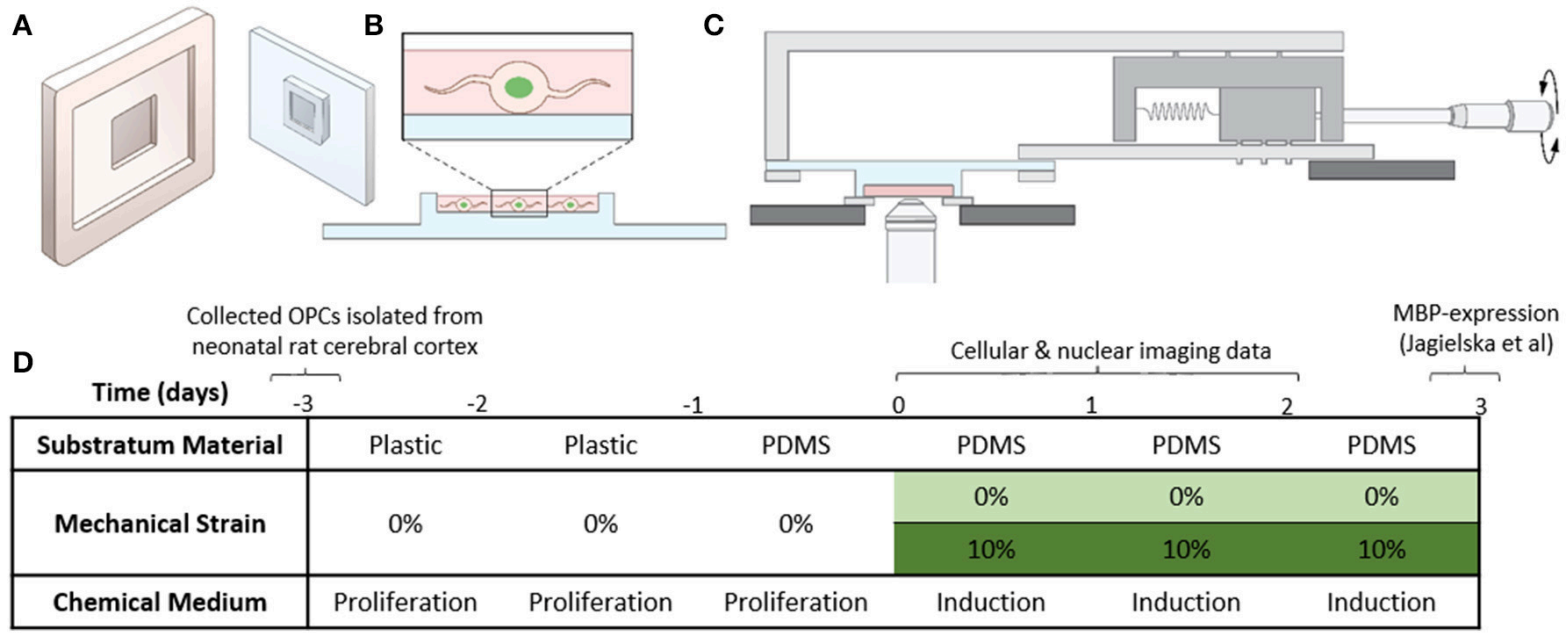
Experimental setup. **(A)** Cartoon of the plastic mold and the PDMS plate. **(B)** OPCs transfected with H2B-GFP are seeded in the thin well on the PDMS plate. **(C)** PDMS plate with cells mounted on the stretcher for imaging on the inverted microscope. **(D)** Table summarizing the experimental conditions (details described in section Cell Culture and Media).

Customized polydimethylsiloxane (PDMS) cell culture plates were fabricated from Sylgard 184 silicone elastomer (Dow Corning), using 20:1 ratio of base to curing agent. The silicone mixture was degassed, poured into molds (Figures 1A,B, Figure S1A), and cured for 2 h at 80°C. Cured plates were removed from molds and soaked in acetone (room temperature, 12 h), to remove unreacted oligomers. After 12 h of drying at 45°C, plates were soaked in ethanol for sterilization (12 h), then dried at 45°C (12 h) and stored under sterile conditions. For cell seeding, PDMS plates were first UV sterilized (thrice for 500 s), and then functionalized with fibronectin (from bovine plasma, Sigma). For functionalization, plates were first activated in oxygen plasma for 5 min to make the silicone surface hydrophilic. This was followed by immediate incubation with APTES for 2 h [(3-Aminopropyl) triethoxysilane, Sigma, 100 mM, room temperature] to introduce –NH_2_ groups to the silicone surface, and followed by three washes with deionized water. Next, the plates were incubated for 4 h at room temperature with a solution of molecular cross-linker BS3 (1 mM, Covachem) and fibronectin (100 μg/ml) in HEPES buffer (50 mM, pH 8.0), followed by three washes with PBS (phosphate buffer saline, pH 7.4). The efficiency of ligand deposition was verified in separate experiments with fluorescently labeled fibronectin (Cytoskeleton). OPCs were seeded on PDMS plates at densities ~35,000 cells/cm^2^.

### Application of tensile strain to cells plated on elastomeric PDMS plates

Before application of strain, OPCs were cultured on functionalized PDMS plates in progenitor media for 24 h, at 37°C and 5% CO_2_, to ensure sufficient cell attachment to the surface. For strain application, we used our custom-designed strain devices (Zeiger, 2013; Zeiger et al., 2016) (Figure 1C, Figure S1B), and applied 10% static tensile strain to OPCs grown on the custom-fabricated and functionalized PDMS plates as described above, for 1, 24, or 48 h. During strain application, cells were incubated in differentiation media, at 37°C, 5% CO_2_. Control samples were OPCs cultured on PDMS plates under the same conditions, but without applied strain.

### Transfection and staining

For fluorescence visualization of nucleus in live cells, we used OPCs transfected with H2B-GFP (histone 2B fused to green fluorescent protein). Briefly, CellLight H2B-GFP BacMam2 mix (Thermo Fisher Scientific) was added to the cell media at 2 μL per 50,000 cells. Time-lapse fluorescence movies of H2B-GFP expressing OPCs were acquired 10–16 h after cell transfection with H2B-GFP construct.

For cytoskeleton fluorescence imaging, cells were fixed with 4% paraformaldehyde for 20 min, washed with PBS, blocked with 1% BSA (bovine serum albumin) in PBS (blocking solution) for 30 min, and permeabilized with 0.1% Triton X100 for 3 min. Primary dye-conjugated antibodies were diluted in blocking solution and incubated with cells at room temperature for 1 h. After three washes with PBS, cell nuclei were stained with Hoechst. For F-actin staining, we used phalloidin conjugated with AlexaFluor-594 (Invitrogen) and for microtubule staining we used mouse anti-tubulin monoclonal antibody conjugated with AlexaFluor-488 (Invitrogen).

### Live and fixed cell imaging

Live cell bright field and fluorescent imaging of OPCs was performed using Olympus FluoView Confocal Microscope (with open pinhole). The strain device that was holding the PDMS plate with cells was mounted upside-down on the microscope stage (Figure 1C) to be able to perform high resolution imaging of the nucleus on the inverted confocal microscope using 100X, 40X, and 20X objectives. A plastic holder with a thin glass-coverslip in the center was used to contain a thin layer of medium between the cells and the objective lens.

For capturing nuclear fluctuations in strained and unstrained OPCs, cells were selected based on morphology in bright-field images (see Figure S2 for complementary marker-based verification of highly enriched OPC populations, and discussion of approach taken to maximize probability that these label-free cells selected for time-lapse imaging were OPCs). Time-lapse fluorescence imaging of H2B-GFP labeled cells was conducted at 30 s interval for total duration of 30 min (total 60 frames), using either 100X or 40X objectives. For comparing nuclear fluctuations of unstrained OPCs to terminally differentiated oligodendrocytes, time-lapse fluorescence imaging of H2B-GFP labeled cells was conducted at 2.5 min interval for total duration of 1 h using 40x objective (total 24 frames). For capturing cell migration, bright field imaging was performed at 36 s interval for total duration of 1 h (total 100 frames), using 20x objective. Images were processed and analyzed using MATLAB and Fiji.

Fluorescently stained F-actin and microtubule cytoskeleton was imaged using an upright confocal microscope (Olympus) with 40x water-immersion objective and 5x digital zoom. Z-stacks in each fluorescence channel were collected with a 0.8 μm step. Images were processed and analyzed in Fiji (Schindelin et al., 2012).

## Image analysis

Post-imaging, based on the bright-field channel, the single-cell movies were manually classified to distinguish and identify those movies that included a cell considered most likely to be an OPC, rather than those unlikely to be OPCs due to morphology or cells that exhibited blebs or morphological characteristics of apoptosis (see Figure S2 and associated discussion). Only those selected movies were analyzed further.

### Nuclear projected area fluctuations

Time-lapse fluorescent images of nuclei captured using open-pinhole were thresholded and the nuclear area, circularity and solidity were calculated using custom-written codes in MATLAB. Next, the nuclear area (in square micrometers) vs. time were plotted and de-trended using a third-order polynomial fitting in ORIGIN. The percentage residual fluctuations were calculated as [residual (*t*)/value of polynomial (*t*)]^*^100 at each timepoint *t*. From the percentage residual fluctuations, the standard deviation and variance were computed to compare the amplitude of area fluctuations in different conditions.

### Circularity fluctuations and edge fluctuations

Circularity was calculated as $R=\frac{4\;*\;area}{\pi *{\left(major\;axis\right)}^{2}}$ where major axis is the length of the primary axis of the best fitting ellipse. For each nucleus, time lapse sequence of fluorescence images, was converted to binary format via grayscale thresholding and the average (μ) and standard deviation (σ) of circularity was calculated over time sequence. Circularity fluctuations were calculated for each nucleus as scaled standard deviation $S=\frac{\sigma }{\mu }\;*\;100\%$ (i.e., coefficient of variation) of time sequence. Edge fluctuations were calculated as non-overlapped area between nuclei at time (*t*) and (*t*+ Δ*t*) divided by total area of nucleus at time *t*.

### Intensity correlation coefficient

Image intensities were first scaled by dividing each pixel intensity by the sum of the intensities of all pixels within the nucleus. Images were manually cropped to 100 × 100 pixel. Alignment of nuclei in the time series was conducted in imageJ using the plugin *Registration* → *Linear stack alignment*. The intensity correlation coefficient was calculated as $C=\sum_{n=2}^{end}\frac{\sum_{i}\sum_{i}\left(x_{1}{}_{i,j}\;-\;x_{1}{}_{ave}\right)*\left(x_{n}{}_{i,j}\;-\;x_{n}{}_{ave}\right)}{\sigma_{1}\;*\;\sigma_{n}}$ where *x* is intensity value, n is time point, σ is standard deviation of intensity of the whole nucleus, and *i* and *j* are the coordinates of a pixel. The correlation coefficient *C* can have values between 0 and 1 with 0 being no correlation and 1 being perfect correlation. The final value of *C* for each time was taken as the average for entire cell population.

### Time stacking (kymographs) and manual tracking

Bright-field image stacks were first aligned in FIJI using the plugin *Registration* → *Linear Stack Alignment*. The aligned stacks were then z (time)-projected using minimum intensity. Migrating cells appeared as streaks of dark cell outlines; streaks that were typically longer than the cell's characteristic length scale were then measured manually. Trajectories that were not identifiable as cell migration (e.g., too short in duration) were not included in further migration analysis. Histograms of trajectory lengths were graphed using ORIGIN.

### Nucleus trajectory

Nucleus centroid coordinates were calculated from the thresholded nucleus images, centroids of consecutive frames were connected with straight lines, and an image of all straight lines to show nucleus trajectory was generated, using custom-written code in MATLAB. The length of the nucleus trajectory was measured manually while correcting for any drift observed from the corresponding bright field movie. It should be noted that there are differences between the nuclear-centroid-tracking method and the time-stacked image analysis method for measuring cell migration: (1) shorter field-of-view in 40x fluorescent movies of nucleus compared to 20x bright field movies, (2) shorter duration, 30 min, of fluorescent movies of nucleus compared to an hour for low-zoom bright field movies, and (3) nucleus-centroid tracking also includes small-scale cell dynamics such as cell-detachment or cell-spreading, the contribution of which to trajectory length is typically much less than the characteristic length of a cell.

#### Sample size and statistical tests

Hundreds of selected cells/movies were analyzed to further minimize noise from potential cell population impurities (see Figure S2). For nuclear fluctuations data, 200 high resolution movies (40X/100X) of single cells with H2B-GFP tagged nuclei were acquired; the cell migration data was obtained from manual tracking of 180 cells from bright field time-stacked images (40X/20X). One way ANOVA with Bonferroni correction was used to determine whether the differences observed were statistically significant.

## Results

### Mechanically strained OPCs exhibited hastened dampening of nuclear fluctuations during differentiation

Dampened or decreased fluctuations of the nucleus projected area (i.e., nucleus shape or outline) have been correlated previously with embryonic stem cell differentiation (Pajerowski et al., 2007; Bhattacharya et al., 2009; Talwar et al., 2013), and with restricted potency of mesenchymal stem cells (Lee et al., 2014). We also observed herein that terminally differentiated oligodendrocytes exhibited significantly lower nuclear fluctuations than undifferentiated OPCs (Figure S3). However, beyond that binary comparison at uncommitted and committed timepoints, here we sought chiefly to compare nuclear fluctuations and subcellular dynamics of OPC cells upon response to differentiation induction, with and without application of tensile strain.

**Figure 2:**
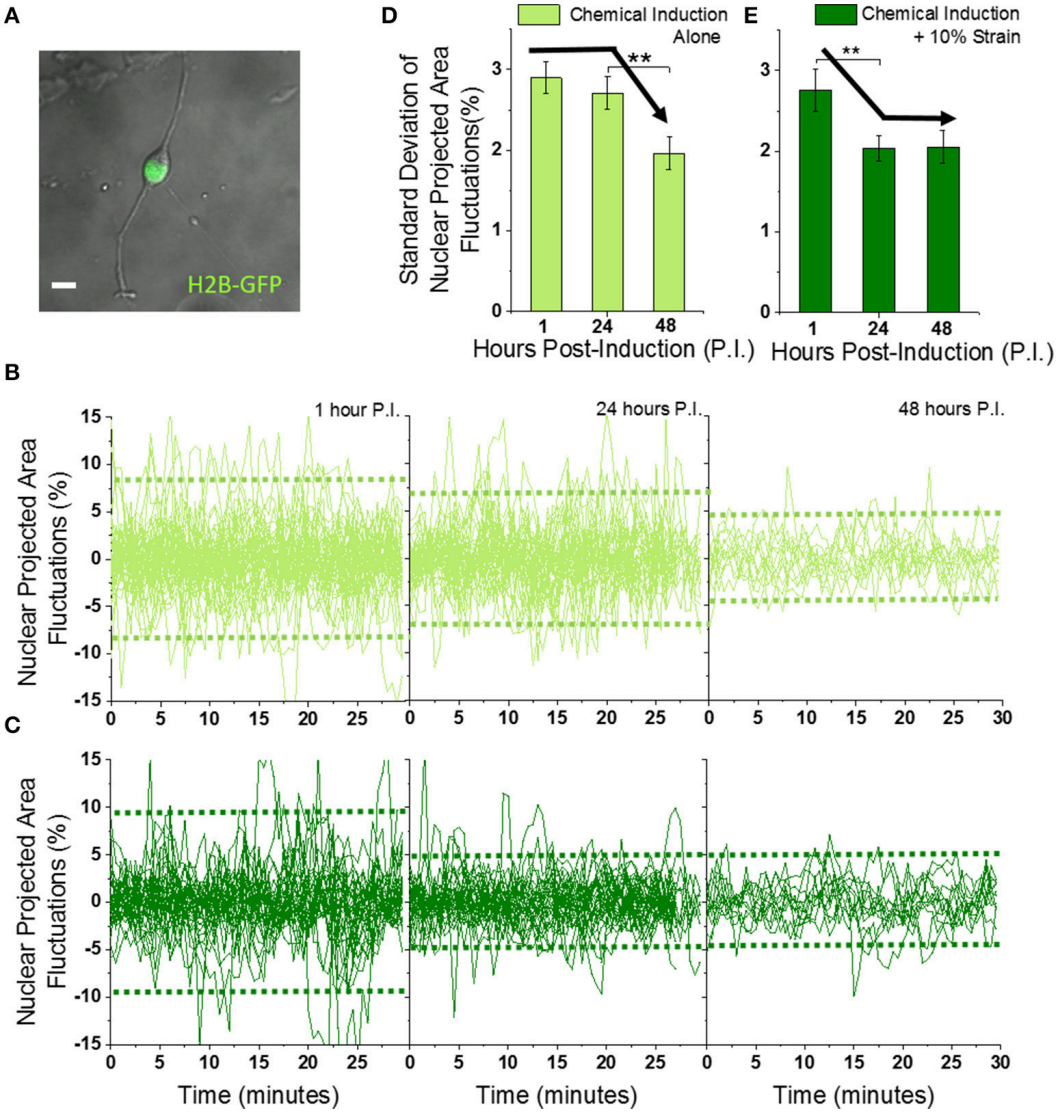
Mechanically strained OPCs exhibit faster dampening of Nuclear Projected Area Fluctuations during differentiation. **(A)** Typical image of OPC showing cell body and processes in the bright field (gray), and nucleus in the GFP fluorescence (green) captured using 40X objective. Scale bar 10 μm. **(B,C)** Fluctuations in nuclear projected area, calculated from time-lapse imaging (60 images at 30 s interval) of OPCs undergoing differentiation without **(B)** or with **(C)** 10% strain, for 1, 24, and 48 h post-induction (see Figures S4A,B for calculation of area fluctuations). Dotted horizontal green lines represent average variance of the fluctuations. Unstrained *n* = 59 (1 h), 46 (24 h), 13 (48 h); Strained *n* = 38 (1 h), 35 (24 h), 12 (48 h). **(D,E)** Standard deviations of time series plotted in **(B,C)** to compare amplitude of nuclear area fluctuations. Solid black arrow lines drawn manually to highlight the differential decreasing trend without and with 10% strain. Error bars represent standard errors. ^**^p < 0.05.

We isolated OPCs from neonatal rat cerebral cortex and cultured cells in proliferation medium on standard tissue culture polystyrene dishes for 2 days, before transferring them to fibronectin-coated PDMS plates (see section Materials and Methods). After 24 h of cell attachment on PDMS in proliferation medium, we exchanged media to replace with SATO oligodendrocyte differentiation medium and immediately applied 10% static strain (Figure 1D, section Materials and Methods); here, the term static indicates that the applied strain was maintained constant over the duration of the experiment. Thus, we initiated chemical induction of differentiation (i.e., exchange of culture media composition), and provided concurrently the presence or absence of a mechanical cue. We conducted live-cell time-lapsed imaging of strained and unstrained cells at 1, 24, and 48 h post-induction to measure nuclear and cellular dynamics that precede the strain-mediated upregulation of myelin basic protein, which was observed 3–5 days post-induction in our previous work (Jagielska et al., 2017). We quantified nuclear dynamics using cells transfected with fluorescently-tagged histone H2B-GFP ~12 h prior to imaging (Figure 2A, section Materials and Methods), imaged every 30 s over a duration of 30 min for each cell. We then analyzed these images to obtain nuclear projected area fluctuations as a function of time (see section Materials and Methods).

The amplitude of nuclear fluctuations was ~3% at 1 h post-induction, in both unstrained and strained OPCs when quantified as standard deviation (or 9% expressed as variance) (Figures 2B–E, Figures S4A,B, and Supplementary Movie 1). The area fluctuations of strained cell nuclei decreased to 2% (or 4% expressed as variance) at 24 h and maintained this magnitude at 48 h post-induction. In contrast, this reduction of nuclear fluctuations was delayed until 48 h post-induction in unstrained cells. These data demonstrate that application of static strain to OPCs under chemical induction enhances a known biophysical marker of differentiation: dampening of nuclear fluctuations.

Nucleus size as quantified by average nuclear area did not change significantly, but nucleus shape quantified by average nuclear circularity decreased upon application of strain (Figures S4C–E). Thus, mechanical strain predictably changed the nucleus shape, and more importantly hastened the dampening of nuclear fluctuations such that these dynamics were reduced in approximately half the time required of chemical induction alone. That is, the dampening of these nuclear membrane displacements occurred sooner in time (24 v. 48 h) when the cells were under constant tensile strain. Next, we probed the effect of strain on cell migration, another biophysical feature that is known to decrease in extent upon oligodendrocyte differentiation, and tested for correlation of this feature with nuclear fluctuations.

### Mechanical strain decreased migration of OPCs undergoing differentiation

OPC differentiation is associated with decreased cell migration: increasingly branched OPCs cease migrating as they differentiate to oligodendrocytes (Small et al., 1987; LeVine and Goldman, 1988; Noble et al., 1988; Reynolds and Wilkin, 1988; Armstrong et al., 1990; Milner et al., 1996). Here, we compared migration trajectory distances of OPCs under chemical induction, with and without applied strain at 1, 24, and 48 h post-induction, to probe whether strain correlated with branched cell morphology and reduced cell migration that would also be consistent with progression of OPC differentiation.

**Figure 3:**
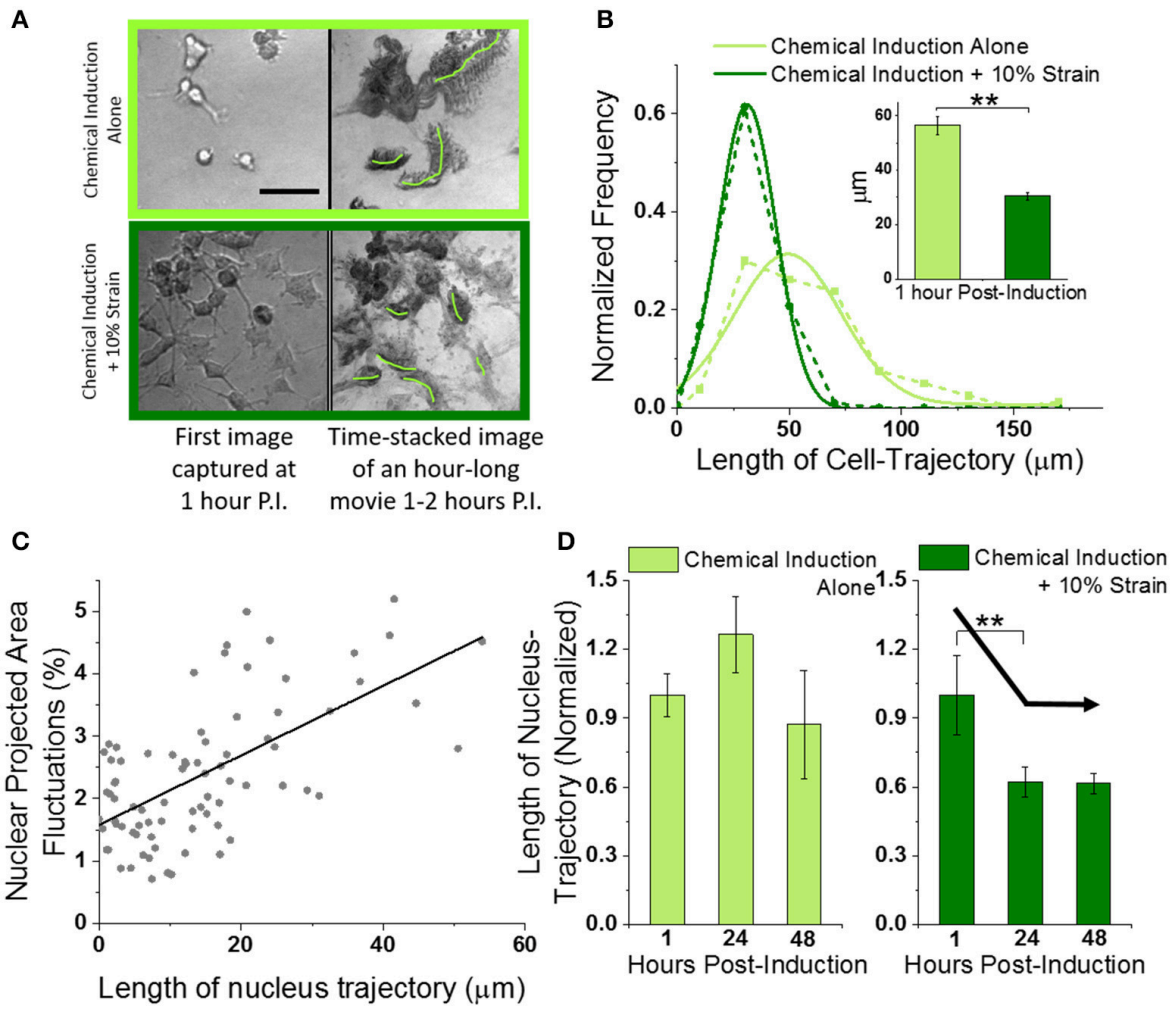
Mechanical strain decreases migration of OPCs undergoing differentiation. **(A)** Left column shows typical field-of-view images of OPCs undergoing differentiation without (top row) or with 10% strain (bottom row), at 1 h post-induction (using 20X objective). Scale bar 50 microns. Right column shows corresponding time-stacked images obtained using minimum-intensity-projections of time lapse images (100 images at 36 s interval) captured starting at 1 h post-induction. Green lines drawn manually in ImageJ to mark trajectories of single cells. **(B)** Histogram of whole-cell-trajectory lengths manually measured from time-stacked images of hour-long bright-field movies (from 1 to 2 h post-induction) without and with 10% strain. Unstrained *n* = 80; Strained *n* = 101. Dotted lines show experimental data while solid lines show Gaussian fits. Inset shows the mean values. Error bars represent standard error. ^**^p = 2E-13. **(C)** Scatter plot of nuclear area fluctuations vs. nuclear trajectory length (both measured over 30 min duration time-lapse images of H2B-GFP labeled OPC nuclei). Gray dots represent combined data from unstrained [*n* = 25 (1 h), *n* = 20 (24 h), *n* = 11 *(*48 h)] and strained [*n* = 15 (1 h), *n* = 7 (24 h), *n* = 9 (48 h)] OPCs. Extreme data points with nuclear trajectory length or nuclear fluctuations amplitude higher than mean + 2^*^S.D. were considered outliers and removed before linear fitting (Figure S8). Solid black line shows linear fit (Pearson's *r* = 0.63). **(D)** Average normalized nuclear-trajectory length plotted as a function of time post-induction. Data has been normalized to average nuclear-trajectory length in unstrained or strained OPCs at 1-h post-induction respectively. Solid black arrow lines drawn manually to highlight the decreasing trend with 10% strain. ^**^p = 0.06.

We measured trajectory distances from time-lapse imaging acquired over 1 h duration at low magnification (Supplementary Movie 2, 20x), using minimum-intensity time-projection (see section Materials and Methods). Interestingly, the mean migration trajectory of unstrained cells in differentiation medium was 60 μm at 1 h post-induction, while that of strained OPCs in same medium was 50% shorter (Figures 3A,B). However, migration trajectories of OPCs in both “*Proliferation”* media and “*Proliferation* + *10% Strain”* conditions were significantly shorter than those in differentiation media, and were so short (<5 μm in 1 h of time-lapse imaging) that we could not manually trace the trajectory from the time-stacked bright-field images (compare the four different rows of Figure S5, where only the last two rows are in proliferation media). We therefore concluded that these mechanical strain experiments in proliferation media (not differentiation media) require future additional analysis related to cell migration. Interestingly, we observed that strained OPCs in proliferation media exhibited processes with appreciably more dynamic displacement: cell processes extended and retracted from the cell body more often in the strained cell populations (Figure S6, Supplementary Movie 4).

Since both nuclear fluctuations and cell migration are biophysical markers of cell dynamics, we investigated whether hastened dampening of nuclear fluctuations in strained cells was correlated with reduced cell migration. Hence, we also tracked the nucleus centroid from the same (40x) fluorescent movies of OPC nuclei that were used for measuring nuclear fluctuations at 1, 24, and 48 h post-induction (Figure S7, Supplementary Movie 3, see section Nuclear Trajectory). The nuclear membrane fluctuations were correlated strongly with nuclear trajectory length (Figure 3C, correlation coefficient of 0.6). Thus, both the cell migration inferred from nuclear trajectory lengths and the nuclear membrane fluctuations were reduced at earlier timepoints in strained OPCs as compared to unstrained cells (Figure 3D).

### Mechanical strain increased production of microtubule cytoskeleton

**Figure 4:**
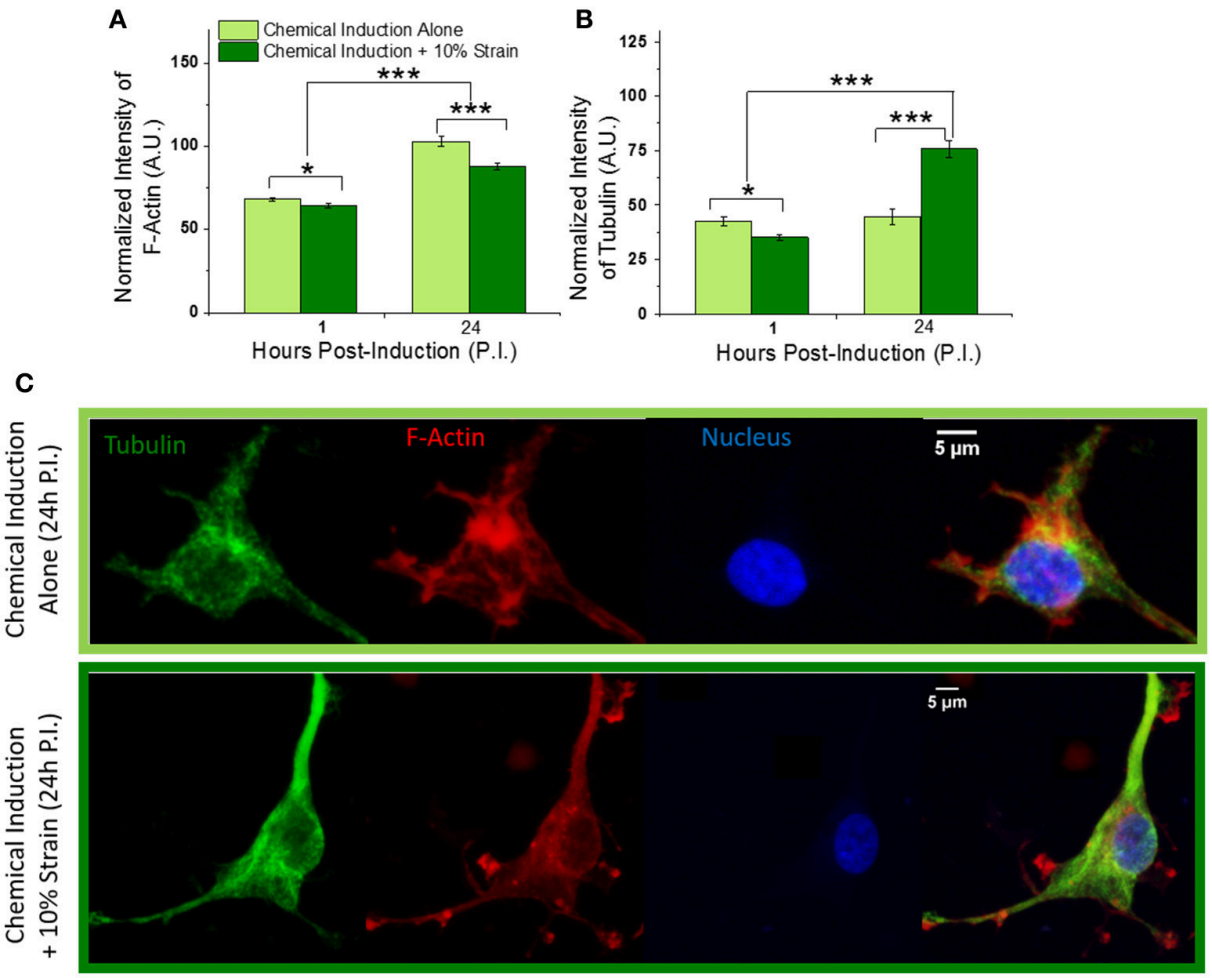
Effect of mechanical strain on OPC cytoskeleton. **(A)** Total fluorescence intensity of F-actin and **(B)** tubulin normalized by actin and tubulin volume, respectively, at 1 and 24 h post-induction of differentiation, for strained and unstrained OPCs. F-actin levels increase to similar extent for strained and unstrained cells with differentiation time progression from 1 to 24 h; F-actin expression is reduced in response to strain, by 6 and 15% at 1 and 24 h post induction, respectively. Tubulin levels increases by 70% in strained cells at 24 h time point. **(C)** Examples of F-actin (red) and microtubule (green) cytoskeleton in unstrained (top) and strained (bottom) cells at 24 h time point; Hoechst nuclear staining in blue. Scale bar 5 μm. *N* = at least 20 cells per condition. Error bars are SEM (standard error of the mean); ^*^p < 0.05, ^***^p < 0.001.

OPC differentiation is also accompanied by changes in cytoskeletal structure as the cell shape changes from a bipolar to increasingly branched, non-migratory morphology (Song et al., 2001). We therefore analyzed how mechanical strain affected the OPC F-actin and microtubule networks. Cytoskeletal staining of F-actin and tubulin (see section Transfection and Staining) in strained OPCs and unstrained controls at 1 and 24 h time points was used to quantify normalized fluorescence intensities of actin microfilaments and microtubules for each cell (total intensity normalized by F-actin or tubulin volume from 3D confocal z-stacks). Our results indicated increased levels of F-actin at 24 h compared to the 1 h differentiation time point for both unstrained controls and strained cells, consistent with typical progression of OPC differentiation (Song et al., 2001). However, addition of strain reduced relative F-actin levels by 7% at 1 h and by 15% at 24 h, compared to the unstrained cells (Figures 4A,C). This relative reduction agreed with our earlier RNAseq data, which indicated a 16% decrease of actin gene levels in strained cell populations at 24 h (Jagielska et al., 2017). The expression of tubulin initially decreased by 18% in strained cells at the 1 h time point in the present experiments. However, at 24 h post induction we observed a 70% increase of microtubule production in strained cells compared to unstrained controls (Figures 4B,C), consistent with our earlier RNAseq data that showed 54 and 31% increase in tubulin gene levels for TUBB4a and TUBA1a, respectively, at 24 h post induction (Jagielska et al., 2017). This strain-induced increase in tubulin levels corresponded with formation of microtubule bundles in cell processes (extensions) and cell bodies (Figure 4C, Figure S9). Based on the actin and microtubule structure in the cell processes, we classified the cells as exhibiting either “actin-dominant processes” (type-1) or “tubulin-dominant processes” (type-2). Indeed, 78% of the strained population but only 15% of the unstrained population were type-2 cells, which exhibited well developed microtubule bundles and a corresponding decrease of actin content in cell processes. On the other hand, 22% of the strained population and 85% of the unstrained population were type-1 cells, where the microtubule arrangement was more diffuse and actin was the dominant cytoskeleton in cell processes. Over the course of OPC differentiation, microtubules are known to form dense bundles in cell processes (Song et al., 2001). Thus, the strain-induced accelerated development of the microtubule cytoskeletal structure that we observed herein was consistent with that anticipated during differentiation progression, and further confirms our previous results demonstrating elevated tubulin gene expression in strained OPC populations (Jagielska et al., 2017).

## Discussion

Several recent studies have demonstrated that glial cells, similar to other adherent cell types, are mechanosensitive such that cell biology and function depend strongly on extracellular mechanical cues such as substratum or matrix stiffness (Flanagan et al., 2002; Georges et al., 2006; Lu et al., 2006, 2010; Saha et al., 2008; Kippert et al., 2009; Christ et al., 2010; Moshayedi et al., 2010, 2014; Franze et al., 2011, 2013; Jagielska et al., 2012; Franze, 2013; Lourenço et al., 2016; Urbanski et al., 2016) and applied stresses or strains (Bray, 1979; LaPlaca et al., 1997, 2005; Cullen et al., 2007; Franze et al., 2009, 2013; Franze and Guck, 2010; Lindqvist et al., 2010; Betz et al., 2011; Ahmed et al., 2013; Arulmoli et al., 2015; Hernandez et al., 2016; Poitelon et al., 2016; Jagielska et al., 2017; Shimizu et al., 2017). Mechanical strain in the central nervous system can originate from various physiological/developmental processes such as developmental tissue reorganization, fluid flow, and axon growth, as well as pathological processes including axon swelling or mechanical trauma. We have shown previously that mechanical strain within a physiological range (tensile strain of 10% magnitude) can promote differentiation of oligodendrocytes in terms of number of MBP+ cells at a given time point, and is associated with altered chromatin organization and gene expression (Jagielska et al., 2017). As oligodendrocyte differentiation is required for myelination and correct neurological functions, strain-driven differentiation has implications for understanding myelin formation and repair.

Recent studies have explored the role of various molecular components involved in mechanotransduction of specific physical cues during oligodendrocyte differentiation. We have demonstrated that specific interactions of cell surface receptors with extracellular matrix ligands such as fibronectin and laminin are essential for mechanotransduction of applied strain, and its effects on OPC differentiation and proliferation (Jagielska et al., 2017). Integrin-laminin engagement is critical for strain-mediated differentiation of neural progenitor cells into oligodendrocytes (Arulmoli et al., 2015). The activation of integrin signaling pathways, specifically including the Src-family kinases Fyn and Rho, Rac, and Cdc42 GTPases, governs cytoskeleton remodeling during morphological changes associated with oligodendrocyte differentiation (Bauer et al., 2009). Efficiency of oligodendrocyte differentiation can also be varied with substratum stiffness (Jagielska et al., 2012); sensing of matrix stiffness by oligodendrocytes involves actomyosin contractility (Kippert et al., 2009) and is dependent on non-muscle myosin II (Urbanski et al., 2016). The transcriptional activator YAP, which is known to be responsive to cytoskeletal dynamics, shuttles to the nucleus in strained OPCs (Shimizu et al., 2017). Further, the nesprin-1 protein in the LINC complex enables force transduction to the nucleus for downstream epigenetic changes and chromatin remodeling in oligodendrocytes (Hernandez et al., 2016). We showed previously that mechanotransduction of static strain in rat OPCs proceeds within the nucleus to decrease histone acetylation (via HDAC11) and to confer global changes in gene expression that are consistent with oligodendrocyte differentiation (Jagielska et al., 2017). However, the biophysical state of OPCs during the early stages of this mechanical response, specifically in terms of cellular and nuclear dynamics during differentiation, was not addressed. Additionally, those time intervals of observations were days (not hours), due to the typical *in vitro* timeline of chemically induced differentiation assessment through MBP production and morphology changes at 3–5 days post-induction.

Here, we explored the effect of mechanical tensile strain on oligodendrocyte nuclear dynamics, cell migration, and cytoskeleton during early stages of OPC differentiation, from 1 to 48 h. We report the following novel findings, discussed further in detail. First, tracking of nuclear dynamics in live cells during the first 48 h of OPC differentiation revealed that addition of static mechanical strain dampened nuclear dynamics faster than chemical differentiation cues alone. This dampening effect was not measurable immediately after strain was applied, but instead this nucleus response was delayed by 24 h. The strain dampening effect was effective only for a limited time: after 48 h of maintained strain, there was no longer a differential effect of strain on nucleus dynamics. Second, tensile strain also dampened OPC migration faster than chemical differentiation cues alone, consistent with increased OPC differentiation. Third, tensile strain in OPCs affected mainly the microtubule cytoskeleton, resulting in increased tubulin expression (by 70%); the actin cytoskeleton was modulated to a much lesser extent. The increase of tubulin expression was delayed and observed after 24 h of strain duration, similar to the delayed response of nuclear fluctuations. These findings together elucidate earlier findings that tensile strain accelerates OPC differentiation, and suggest that there could be a specific duration of strain within which key changes in nucleus dynamics occur to promote OPC differentiation.

We showed that physiological magnitude of mechanical strain (10%) hastened dampening of nuclear dynamics, as compared to unstrained cells in otherwise identical biochemical environments. Interestingly, this dampening was not observed immediately after applying strain. Specifically, within 1 h of applying strain there was no detectable change in nuclear dynamics. However, after 24 h of constant strain application we observed significantly greater reduction in nuclear fluctuations. Decreased nuclear membrane dynamics is a signature of cell commitment as cells transition from a stem or uncommitted/progenitor state into specific lineages, attributed chiefly to decreased chromatin reconfigurations upon phenotypic commitment progression (Jaenisch and Bird, 2003; Hubner and Spector, 2010; Milstein and Meiners, 2011; Shivashankar, 2011; Chalut et al., 2012). As we have shown in this work, decreased nuclear dynamics also distinguished fully differentiated oligodendrocytes upon maturation from OPCs in chemically induced differentiation even in the absence of mechanical cues (Figure S3). Therefore, hastened reduction of nuclear dynamics at earlier response time points could be considered as mechanically induced acceleration of differentiation. We further showed that decreased nuclear dynamics correlated with decreased total distances of cell migration, and that decreased cell migration was one result of applied static strain (Figure 3). In other words, mechanical strain resulted in both reduced cell migration distance and reduced nuclear dynamics—and reduction of both cell processes is consistent with oligodendrocyte differentiation progression. Cell migration decreased at an earlier time point under applied strain (at 24 h in strained cells vs. 48 h in unstrained cells), corresponding with the timeline of nuclear fluctuation changes.

These changes corresponded with strain-induced increases in microtubule prominence (total tubulin fluorescence intensity per cell normalized by tubulin volume) and formation of microtubule bundles in cell bodies and processes, observed at 24 h (Figure 4C, Figure S9), consistent with differentiation of OPCs (Song et al., 2001) and with our previous results of increased tubulin gene expression in strained OPCs (Jagielska et al., 2017). The formation of microtubule bundles in cell processes corresponded with simultaneous decrease of F-actin in these processes (Figure 4C, Figure S9). It is notable in that microtubules are connected directly to nuclear lamina via the LINC complex (linker of nucleoskeleton and cytoskeleton) (Roux et al., 2009; Graham and Burridge, 2016) that regulate nucleus shape and position (Wang et al., 2009; Chambliss et al., 2013; Hernandez et al., 2016; Makhija et al., 2016). Thus, though it is beyond the scope of the present study to delineate causal links between the microtubule organization and nuclear membrane fluctuations in OPC response to strain, it is plausible and consistent with other adherent cell types (Holle et al., 2018) that strain is transmitted through the microtubule network to in turn affect chromatin dynamics within the nucleus. Interestingly, the F-actin cytoskeleton responded to tensile strain to a lesser extent than the microtubule elements. Further, F-actin was downregulated in strained OPCs by 15% at 24 h, consistent with our previous RNA sequencing data for the actin gene ACTB at that timpeoint (Jagielska et al., 2017). Notably, Hernandez et al. observed an increase of actin in the nucleus area when OPCs were subjected to acute compressive strain (Hernandez et al., 2016). Although those results cannot be compared directly, it is plausible that these two distinct cues, tensile strain and compressive strain, induce different responses from the cytoskeleton.

An interesting future direction of this study would be to consider whether and how mechanical cues, in the absence of standard biochemical cues of oligodendrocyte differentiation induction, could be sufficient to promote phenotypic commitment. Such a study would require a systematic control of each component of the chemical cues, due to commonality of certain components (T3, T4) in our “differentiation medium” and “proliferation medium,” and presence of an additional cue of growth factors in the “proliferation medium.” Systematic control of each chemical component could elucidate whether tensile strain alone is a sufficient cue to alter cell biophysical response, thereby inducing OPC differentiation while overpowering the inhibiting effect of growth factors. We leave that separate and interesting topic to future studies, to constrain our focus herein on concerted chemical and mechanical cues that achieve more efficient differentiation than currently accessible for these glial cells *in vitro* and *in vivo*.

These results derived from time lapse imaging analysis of OPCs in response to a well-defined mechanical cue, in concert with chemical cues (media composition) known to promote oligodendrocyte differentiation, provide plausible scenarios by which extracellular mechanical strain can promote differentiation of oligodendrocytes. We note that we did not confirm directly that the cells which exhibited strain-induced hastening of damped nuclear fluctuations or reduced migration trajectories also then later differentiated into MBP-producing oligodendrocytes. Such direct tracking of live, unlabeled cells (to minimize perturbation) over the extended durations of such differentiation confirmation (3–5 days) remains challenging, particularly for the high cell numbers required for statistical power of such conclusions. Nevertheless, our findings highlight dynamic connections spanning from the cell-matrix interface to the cytoskeleton to the nuclear membrane, as well as the timeline over which the mechanical signal is transduced to accelerate phenotypic commitment. Specifically, within 1 h of strain application, cells exhibited no detectable response in terms of nuclear fluctuations. That is, the biochemical response to strain was not translated immediately (within 1 h) to the mechanophenotypic signature of damped nuclear fluctuations associated with lineage commitment. After 24 h, however, nuclear fluctuations of strained cells were dampened significantly more than in unstrained counterparts. This hastened dampening of nuclear fluctuations was consistent with increased microtubule network prominence, cell morphology, and reduced cell migration trajectory distances that also represented mechanophenotypic commitment to oligodendrocytes. After 48 h, unstrained cells subjected to only chemical induction finally exhibited the same mechanophenotypic features of oligodendrocyte commitment. This leading response of strained cells, and lagging response of unstrained cells, demonstrated that mechanical cues are transduced by OPCs to accelerate the intracellular and biochemical process of phenotypic commitment. Since strained cells did not show further difference in nuclear fluctuations at 48 h, it would be intriguing to explore the minimum duration of strain required for lineage commitment. However, given that these cells have the potential to form new attachment sites to the substratum while it is under tension, release of that applied tensile strain potentially creates an effectively compressive strain on those cells. Hence, care must be taken in design of such future studies that seek to “switch off” the initial mechanical cue of static tension.

We can now also relate these new observations to prior studies that considered application of this same magnitude of static strain over 3–5 days (72–120 h), which demonstrated that strain resulted in larger number of cells exhibiting the morphology and myelin basic protein (MBP) production of oligodendrocytes, operating through known mechanotransductive pathways (Jagielska et al., 2017). Together, these observations from 1 h to several days post-induction indicate that sustained mechanical strain translates to faster lineage commitment and ultimately to a larger number of oligodendrocytes. Figure S10 shows that strain over short observation time windows in this study approximately (first 24 h of strain) doubled the number of OPCs with damped nuclear fluctuations, and that the same magnitude of strain over longer observation time windows considered previously (72–120 h of strain; Jagielska et al., 2017) doubled the number of cells expressing MBP (as a marker of OL differentiation). Over that same initial 24 h of tensile strain, the same prior study documented concurrent reduction in histone acetylation levels within the nucleus, another corollary of OPC differentiation. In short, extracellular static tensile strain promotes oligodendrocyte differentiation that is plausibly manifested through modulated intracellular dynamics extending all the way to the nucleus and detectable within 24 h. Such understanding can guide future basic and applied research considering mechanical cues or oligodendrocyte-targeted mechanotransductive pathways to promote myelination repair for disease contexts such as multiple sclerosis.

## Conclusions

Extracellular mechanical strain accelerates differentiation progression of oligodendrocyte progenitor cells—a key class of glial cells important to neuron myelination and function—manifested as the onset of biophysical processes consistent with commitment to the oligodendrocyte phenotype. These mechanophenotypic changes include earlier onset of reduced nuclear dynamics and cell migration trajectories, correlated with microtubule cytoskeletal intensity, as tension is transmitted from the cell-material interface to result in altered nuclear dynamics that are not instantaneous but are detectable within 1 day. Quantification of such dynamics at early response time points provide insights into plausible mechanisms by which mechanical strain of physiological magnitude induces intracellular biophysical responses over hours to days that can promote subsequent differentiation of progenitor cells to oligodendrocytes. Understanding of these connections among extracellular mechanical cues, cell migration, cytoskeletal organization and nuclear fluctuations can facilitate basic research of glial cell mechanobiology including myelination, as well as exploration of new engineering cues *in vitro* and therapeutic approaches *in vivo* to stimulate OPC differentiation in nonpermissive disease environments.

## Author contributions

All authors listed have made substantial, direct and intellectual contribution to the work, and approved it for publication.

### Conflict of interest statement

The authors declare that the research was conducted in the absence of any commercial or financial relationships that could be construed as a potential conflict of interest.
