# Supplementary material for: Mechanical Strain Alters Cellular and Nuclear Dynamics at Early Stages of Oligodendrocyte Differentiation
Source: Front Cell Neurosci. 2018 Mar 6;12:59. doi: 10.3389/fncel.2018.00059 (PMC5845683; doi:10.3389/fncel.2018.00059)
Supplement: Supplementary file 5 [file Presentation1.pdf]

Supplementary Figure S1

A B

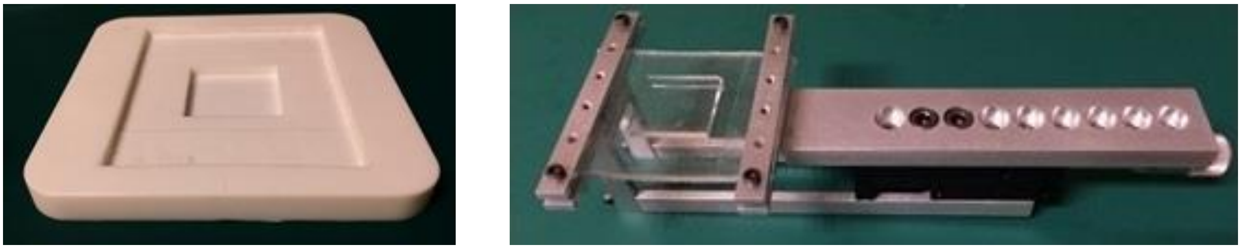

Figure S1: ***Experimental Setup.*** **(A)** Picture of the plastic mold used for making PDMS plates **(B)** Picture of a PDMS plate mounted on the aluminum stretcher

Supplementary Figure S2

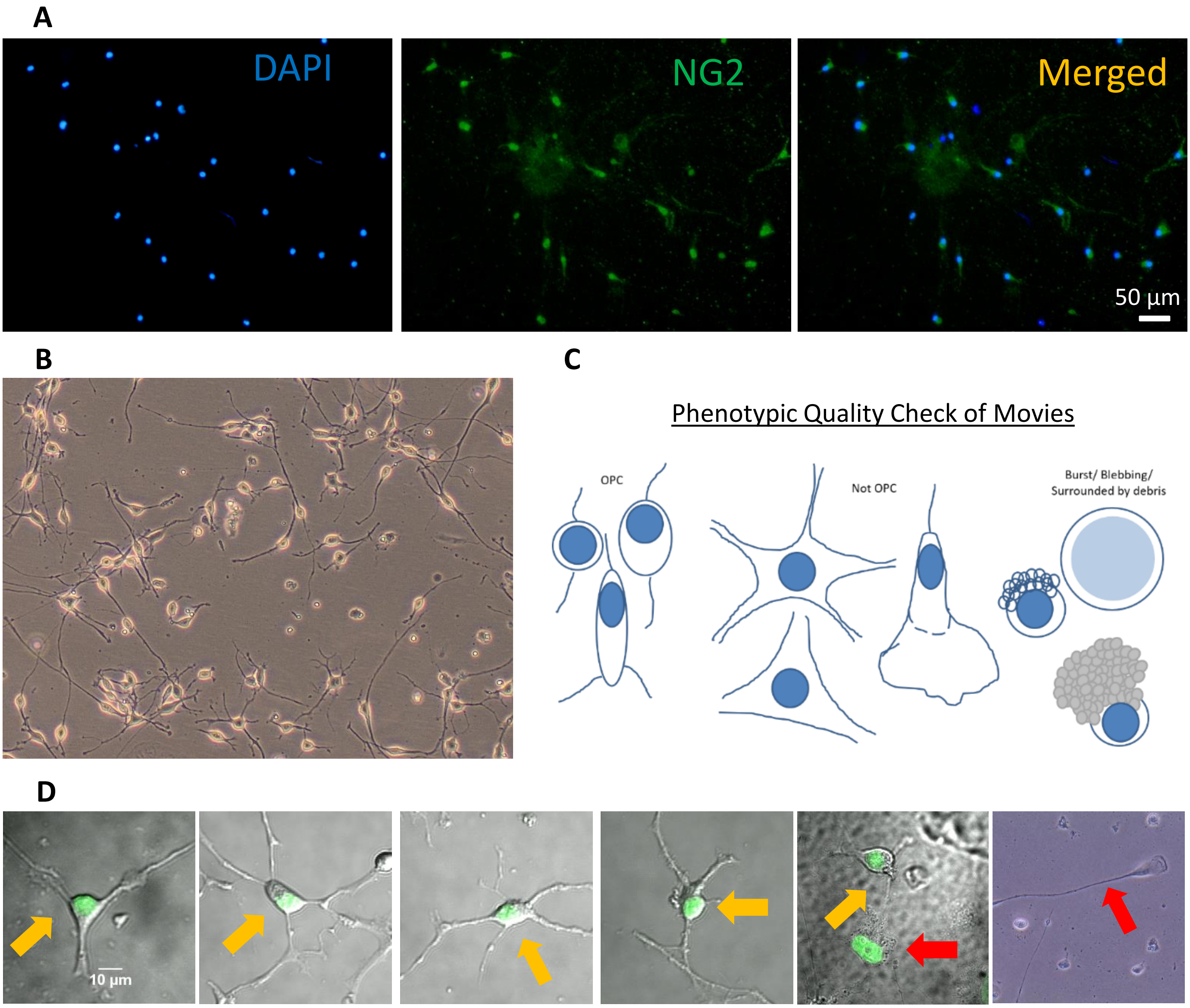

Figure S2: ***Probability of chosen cells being OPC.*** Live-staining against cell surface markers or using cells over-expressing fluorescently-tagged markers introduces additional cell modifications that could potentially alter our observations. Therefore, via the following steps we maximized the probability of chosen cells being OPCs. **(A)** The OPC isolation method yields highly pure OPC population, with OPC content greater than 95%, as quantified by NG2 immunostainings (similar reports by Chen et al 2007, Li et al 2003, McCarthy et al 1980) **(B)** Bright-field images show that majority cells exhibit OPC-like morphology **(C)** Cells were manually screened based on morphology observed in bright field images, before movie acquisition and while analyzing movies post acquisition. Non-OPC-like cells or blebbing cells were discarded. Parameters used to classify a cell as OPC included small round nucleus, small cell body surrounding the nucleus, absence of large lamellipodia-like structures emerging from the central cell body, and at least two processes **(D)** Typical OPC-like (yellow arrow) and non-OPC-like (red arrow) cells. Non-OPC-like cells were not analyzed.

Supplementary Figure S3

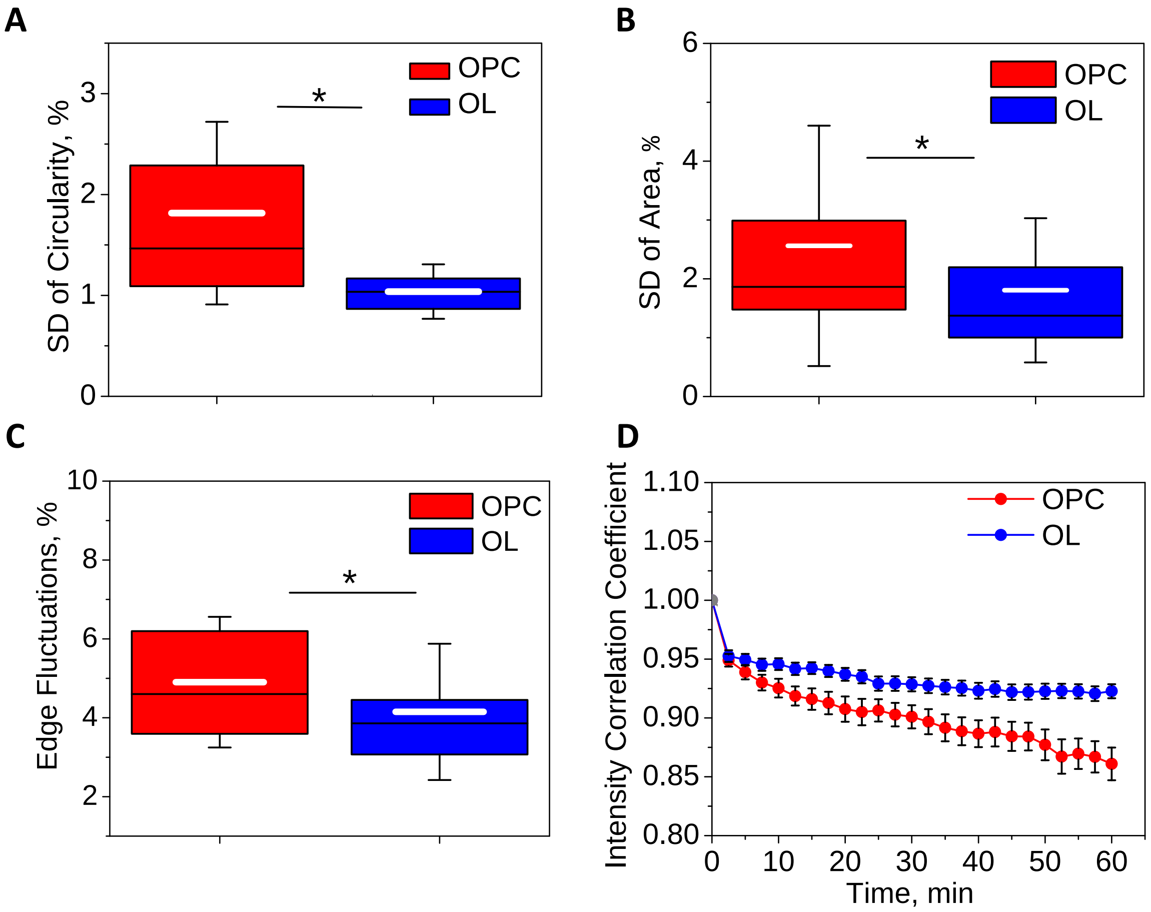

Figure S3: ***Differences in nucleus dynamics between undifferentiated (OPC) and differentiated oligodendrocytes.*** Cells were grown on glass coated with poly-D-lysine, with no applied strain. Nucleus dynamics descriptors were obtained from live fluorescence imaging of nuclei expressing histone H2B-GFP. **(A)** circularity fluctuations; **(B)** area fluctuations; **(C)** fluctuations of nucleus edge; **(D)** correlation coefficient of pixel intensities during observation time of 1 h (see Methods – *Image Analysis 2. Circularity Fluctuations and Edge Fluctuations, 3. Intensity Correlation Coefficient*). Box-and-whisker graphs: box – 25-75 percentile, whisker – standard deviation, black line – median, white line – mean. Blue box – differentiated oligodendrocytes, red box – undifferentiated progenitors (OPC). Error bars are SEM (standard deviation of the mean); * p-value < 0.05. Statistical significance analysis was conducted by one way ANOVA followed by Bonferroni tests. These results collectively demonstrate decreased nucleus dynamics in differentiated oligodendrocytes, reflected in lower fluctuations (A-C) and slower de-correlation of chromatin intensity (D). n=43 (OPCs), 73 (oligodendrocytes)

Supplementary Figure S4

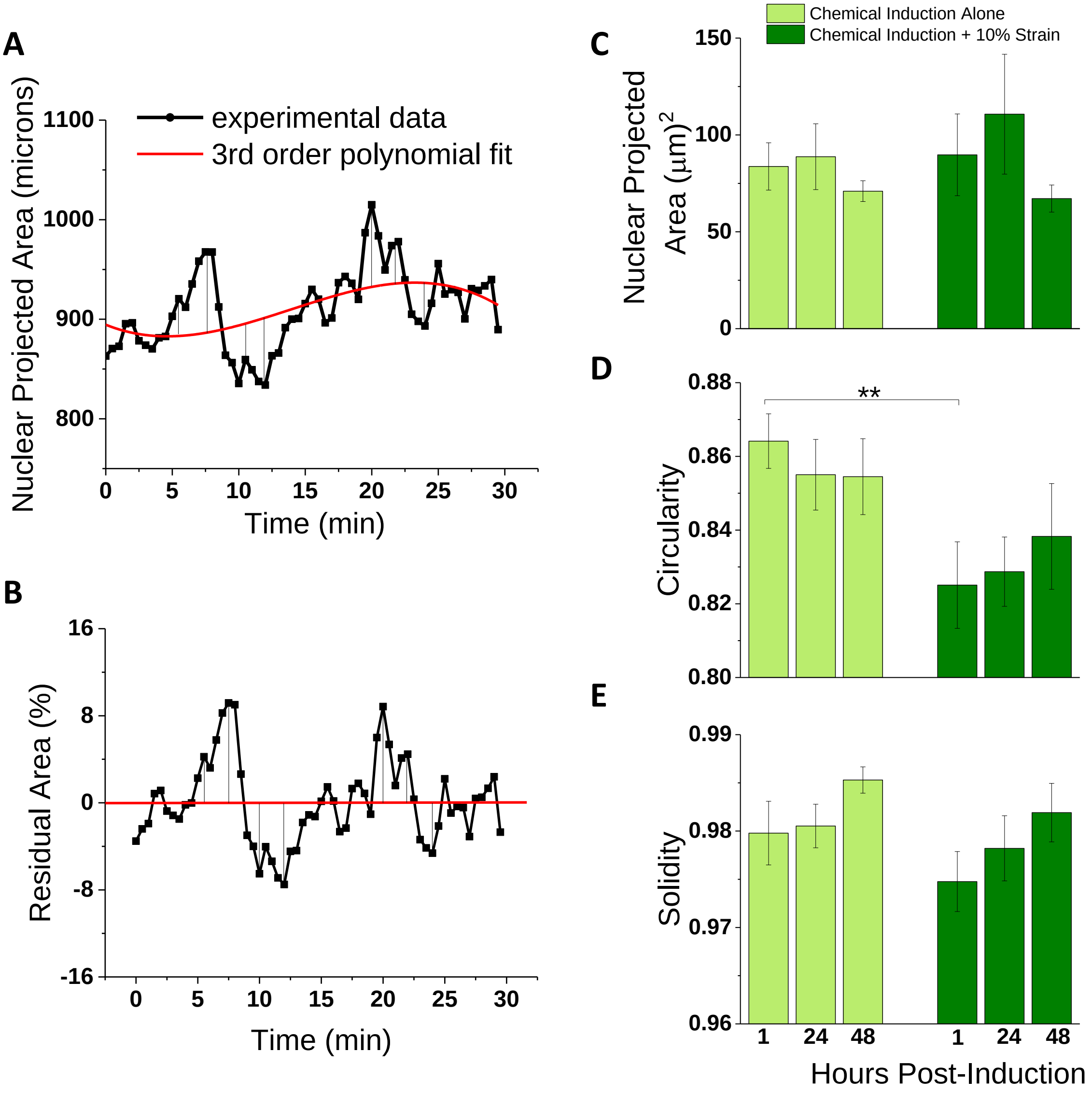

Figure S4: *Mechanically strained OPCs exhibit faster dampening of Nuclear Projected Area Fluctuations during differentiation.* (A-B) De-trending of a typical time-trace of nuclear projected area. Black curve in (A) represents the experimental data for area obtained from thresholded images of the nucleus. Red curve in (A) represents a third-order polynomial fitting. Black curve in (B) represents the percentage residual values obtained from the polynomial fit (details described in *Methods-Image Analysis and Calculations*). Horizontal red line in (B) is shown to highlight that residual fluctuations are centered at zero. Vertical black lines are shown for comparison of peaks and troughs before (A) and after de-trending (B). **(C-E)** Average area, circularity and solidity of the nucleus in unstrained and strained OPC at 1, 24 and 48 hours post-induction (calculated from thresholded nucleus image for only first frame of each movie, not averaged over time). Unstrained n = 59 *(1h)*, 46 *(24h)*, 13 *(48h)*; Strained n = 38 *(1h)*, 35 *(24h)*, 12 *(48h).* Error bars represent standard error. **p=0.003

Supplementary Figure S5

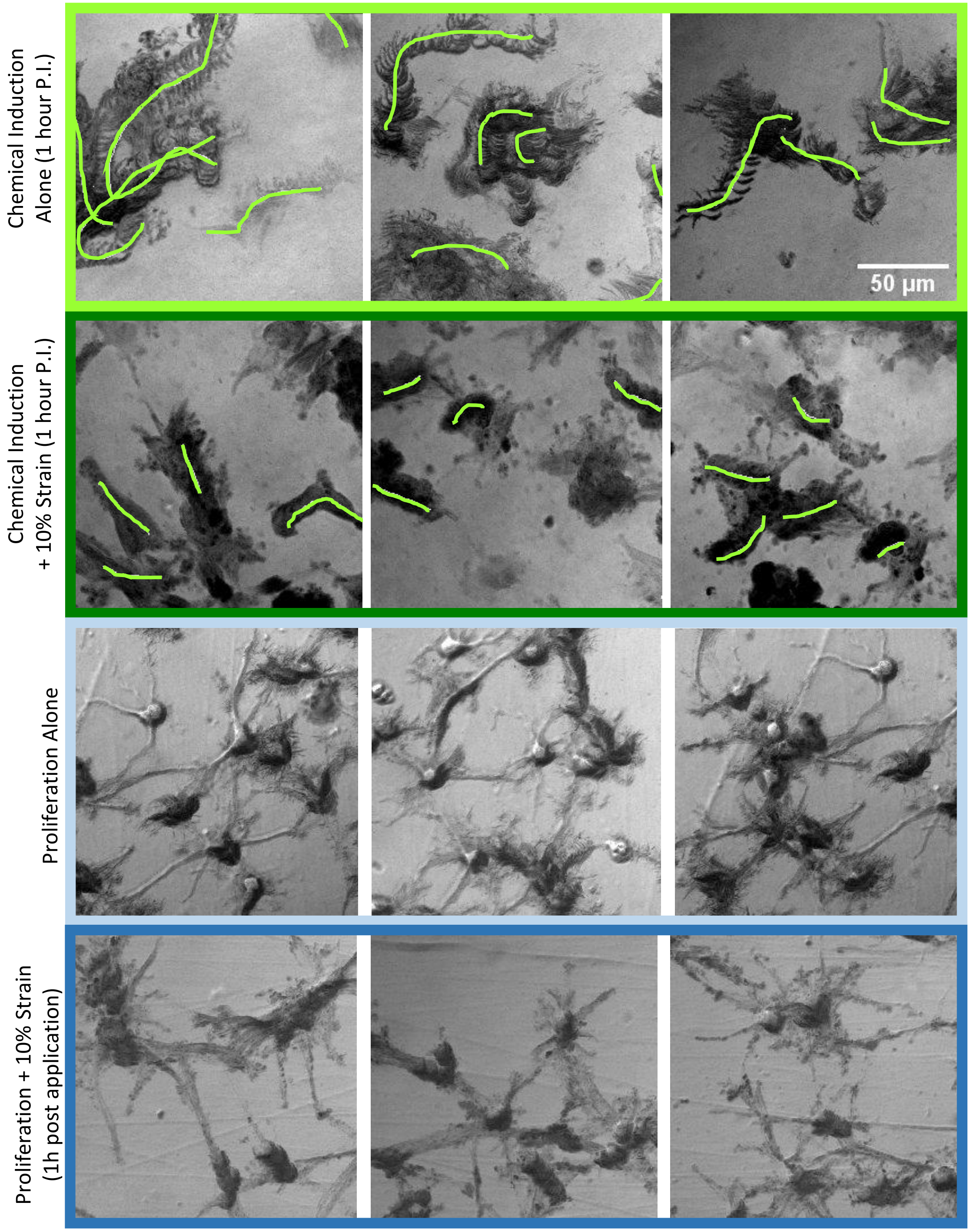

Figure S5: ***Effect of chemical (proliferation/differentiation) and mechanical (+/- strain) cues on cell migration.*** Three typical time-stacked images (obtained from minimum-intensity projections of 100 time-lapse bright field images captured every 36 seconds) of OPCs in chemical induction medium without and with 10% strain at 1-hour post-induction. Bottom two rows show time-stacked images of OPCs before induction, i.e. in proliferation medium, without and with 10% strain. Green curves represent manual tracking of cell-trajectories.

Supplementary Figure S6

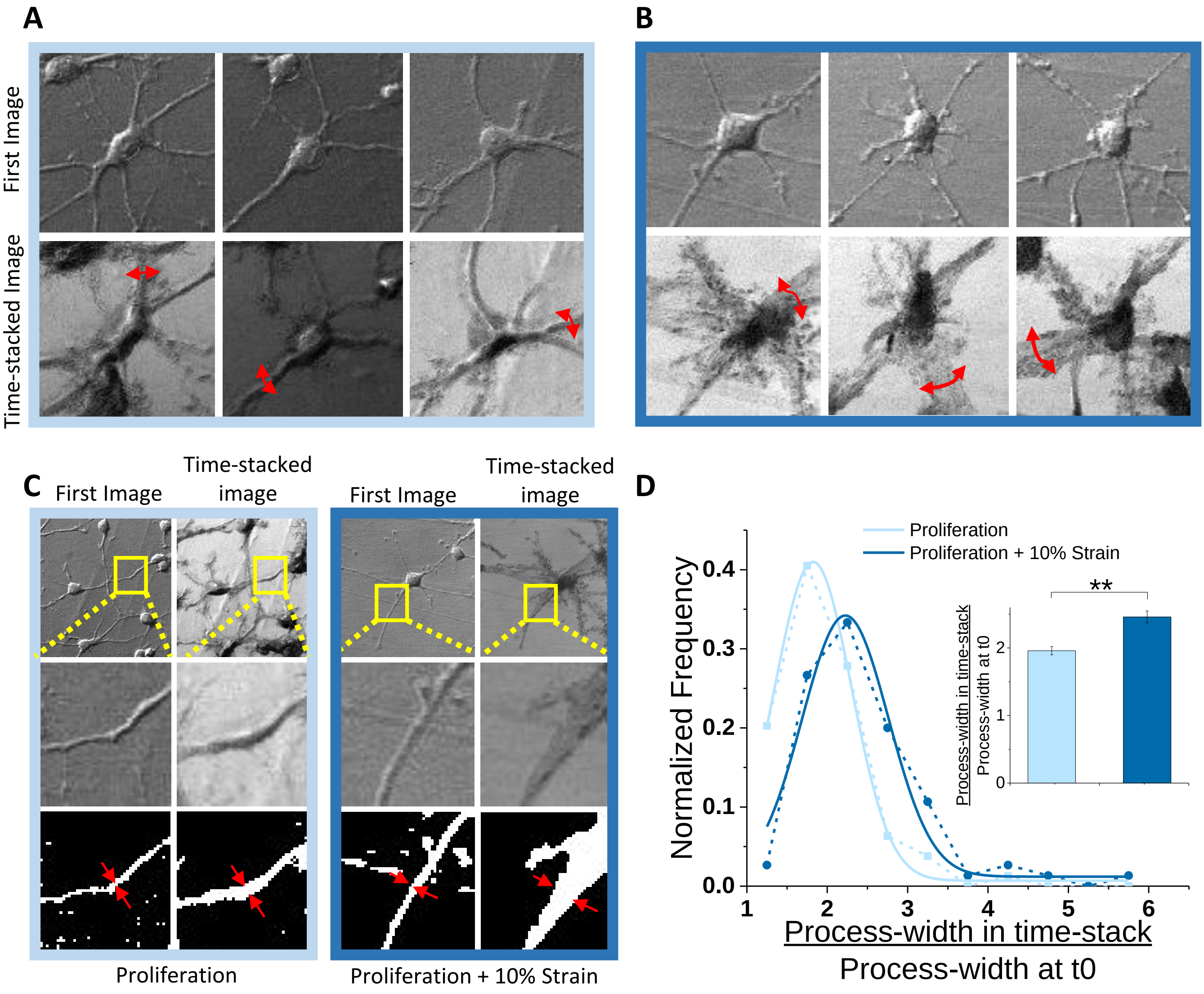

Figure S6: *Greater dynamics of processes in mechanically strained proliferating OPCs*. (A-B) Three typical time-stacked images (obtained from minimum-intensity projections of 100 time-lapse bright field images captured every 36 seconds) of OPCs without (A) and with 10% strain (B) in proliferating condition. Red curved arrows are drawn for easy comparison of process-width in time-stacked images compared to time 0. **(C)** Algorithm for width-measurement in time-stack image and time-0 image **(D)** Histogram of width ratio in time-stacked-image to t0-image without and with 10% strain. Unstrained n = 79; Strained n = 75. Dotted lines show experimental data while solid lines show Gaussian fits. Inset shows the mean values. Error bars represent standard error. **p=6E-6

Supplementary Figure S7

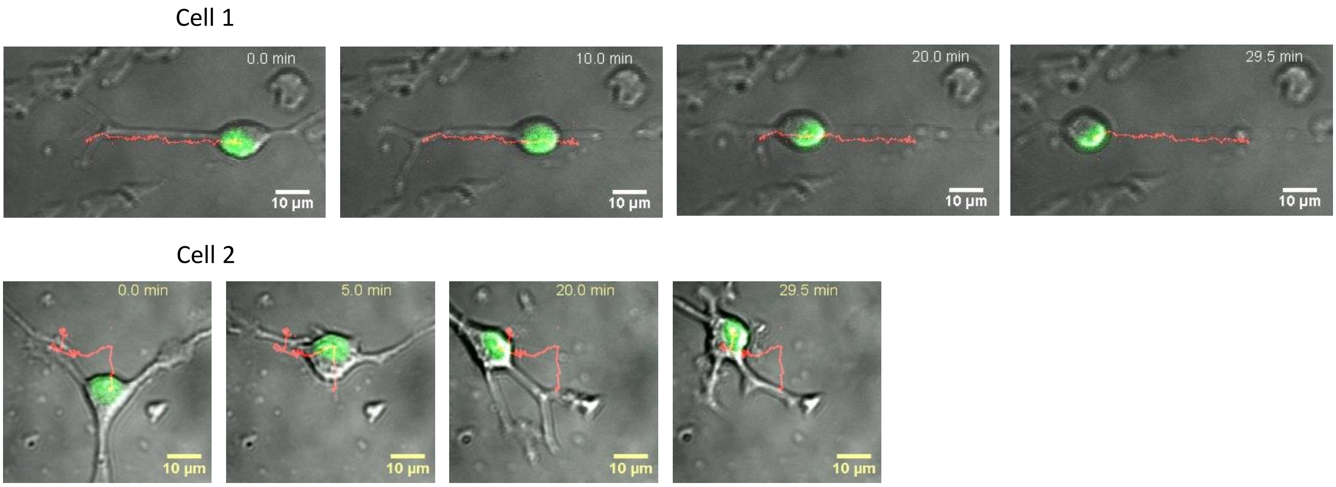

Figure S7: ***Nuclear migration*****.** Snapshots of merge images with bright-field (gray), H2B-GFP expressing nucleus (green) and nuclear-centroid-trajectory (red) from two typical time-lapse image-series of OPCs recorded 1 hour post-induction.

<u>**Supplementary Figure S8**</u>

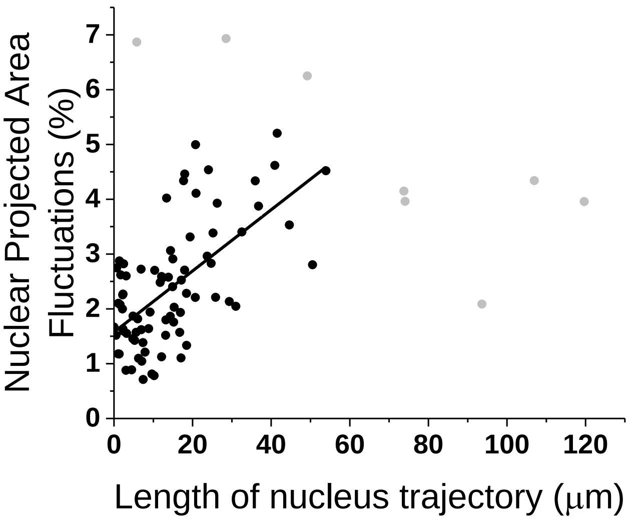

Figure S8: ***Correlation between nuclear projected area fluctuations and nucleus trajectory length*****.** Scatter plot for *87* cells (no strain: 1h-*25*, 24h-*20*, 48h-*11*, 10% strain: 1h-*15* 24h-*7*, 48h-*9*). For the purpose of better fitting, data points beyond mean + 2* S.D. were removed. These are shown in gray. For nucleus trajectory length, mean = 19.45μm, S.D. = 22.65μm, cutoff=64.75μm. For nuclear area fluctuations, mean = 2.6%, S.D. = 1.35%, cutoff=5.3%. Pearson's correlation excluding outliers = 0.63, including outliers = 0.47.

Supplementary Figure S9

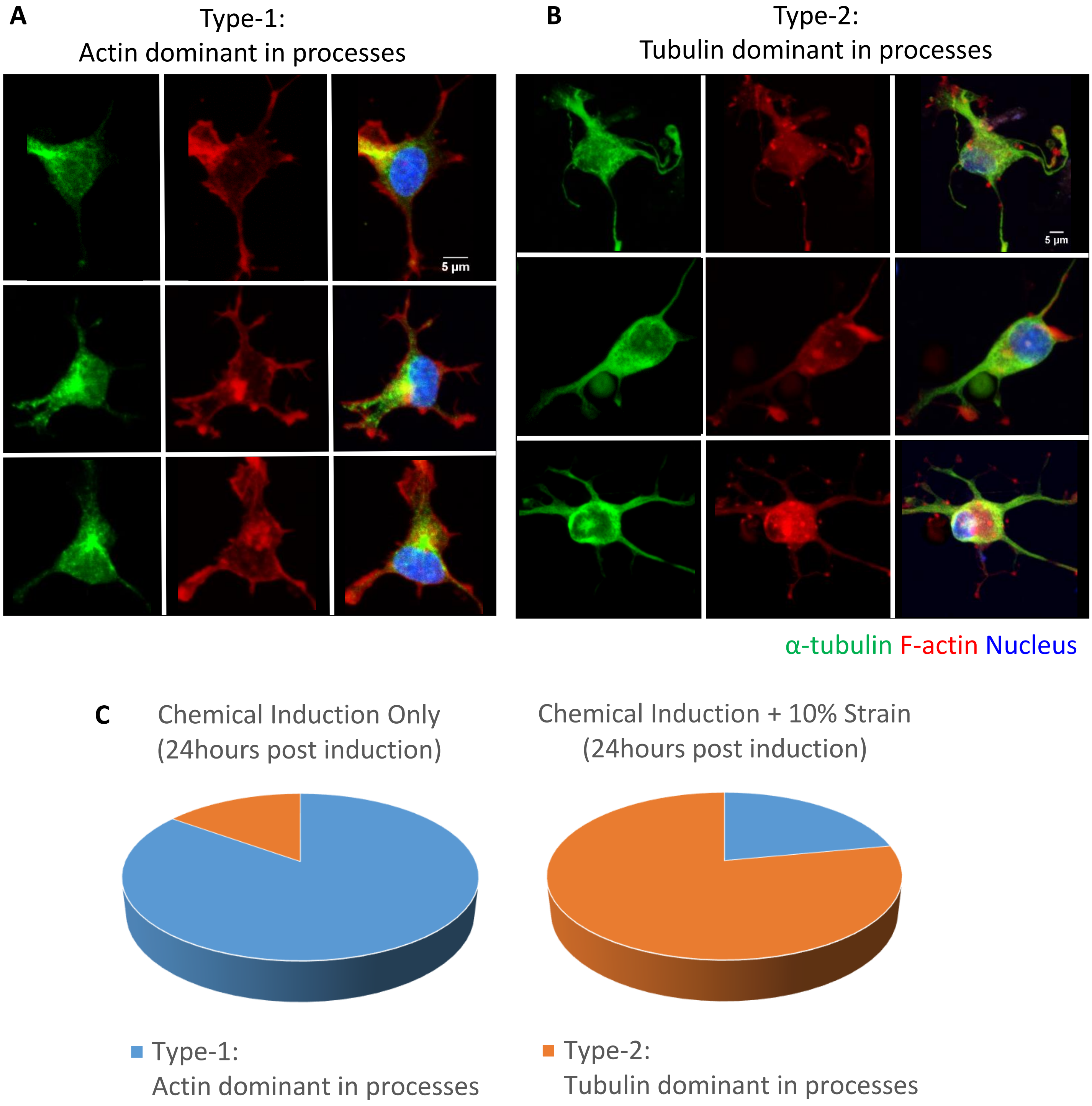

Figure S9: *Differences in cytoskeletal organization*. Representative cells of type-1 (actin dominant in processes) **(A)** and type-2 (tubulin dominant in processes) **(B)**. The brightness and contrast of these images has been adjusted to highlight the differences in cytoskeletal structure. Hence, it would be inappropriate to compare intensities from these images. **(C)** Only 15% of the cells are type-2 at 24h post induction in chemically induced cells, compared to 78% in "chemical induction + 10% strain" condition.

Supplementary Figure S10

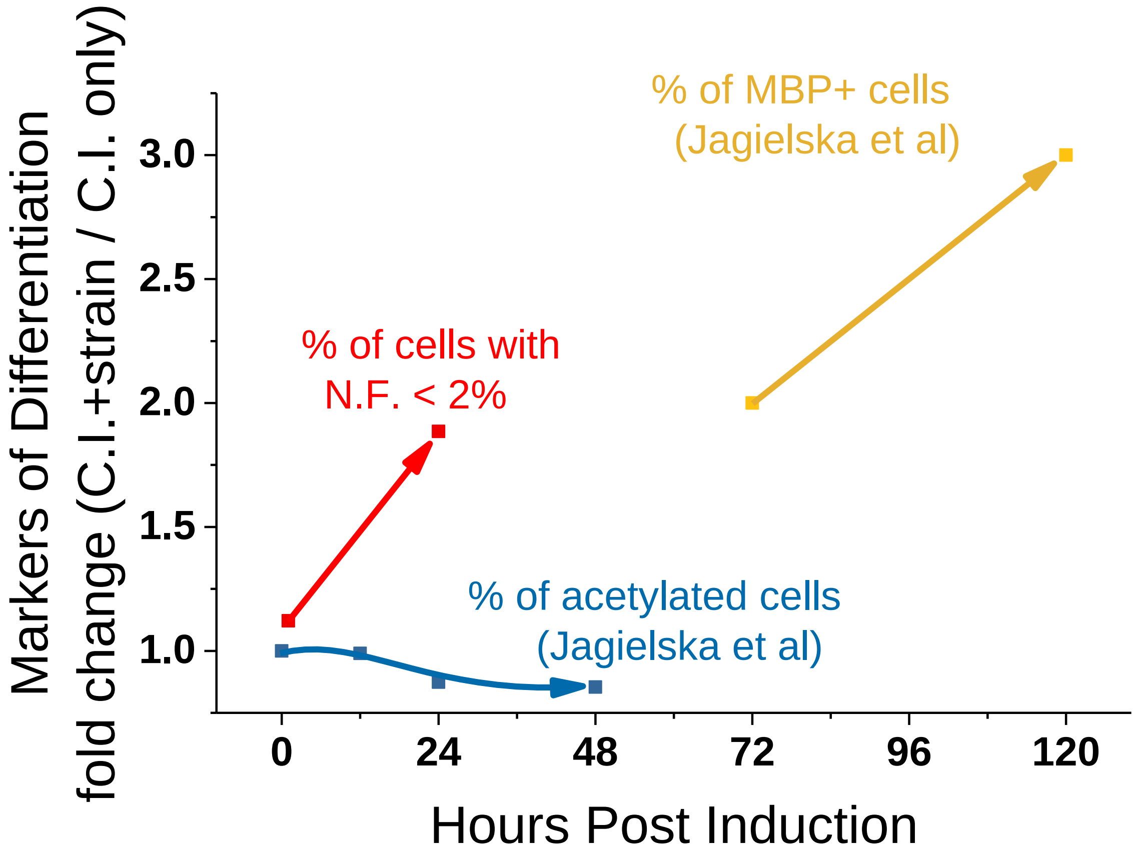

Figure S10: *Temporal event map for various markers of differentiation*. Based on raw data of Fig. 2D, "% of cells with nuclear fluctuations (N.F.) < 2%" were calculated for 1h and 24h post induction for "Chemical Induction (C.I.) Only" and "Chemical Induction (C.I.) + Strain" (red). The threshold value of 2% was chosen since that is the mean amplitude of N.F. at 48h in both conditions. The quantity "% of cells with N.F. < 2%" was not plotted for 48h due to low statistics in that condition. The quantities "% of acetylated cells" (blue) and and "% of MBP+ cells" (yellow) were taken from Jagielska et al. (2017), which were calculated from images of differentiating OPCs stained with antibodies against AcH3K14 (acetylation of lysine 14 on histone H3) and MBP (myelin basic protein), respectively.

Supplementary Movie Legends

Supplementary Movie 1: **Nuclear fluctuations (100X).tif**
Typical time-lapse fluorescence images of nuclei from unstrained and strained OPCs at 1, 24 and 48 hours post-induction. Red outline of nucleus has been generated using custom-written code in MATLAB. Time between frames is 30 seconds. Total duration is 30 minutes. Images were captured using 100X objective.

Supplementary Movie 2: **Cell trajectory (20X).tif**
Typical time-lapse bright-field images of unstrained and strained OPCs at 1 hours post-induction. Time between frames is 36 seconds. Total duration is 1 hour. Images were captured using 20X objective.

Supplementary Movie 3: **Nuclear trajectory (40X).tif**
Typical time-lapse images of unstrained and strained OPCs at 1 hour post-induction. Time between frames is 30 seconds. Total duration is 30 minutes. Images were captured using 40X objective. Bright-field (gray), H2B-GFP labelled nucleus (green) and centroid-trajectory (red).

Supplementary Movie 4: **Process Dynamics in Proliferation + 10% Strain.tif**
Typical time-lapse bright-field images of unstrained and strained OPC under proliferation condition. Time between frames is 30 seconds. Total duration is 30 minutes. Images were captured using 40X objective.
